# Catalyst for good: in Memoriam Mathilde Krim

**DOI:** 10.1002/jia2.25086

**Published:** 2018-02-22

**Authors:** Kenneth H Mayer

**Affiliations:** ^1^ Fenway Health The Fenway Institute Boston MA; ^2^ Department of Medicine Beth Israel Deaconess Medical Center Boston MA; ^3^ Department of Medicine Harvard Medical School Boston MA

**Keywords:** Mathilde Krim, amfAR, AIDS research, AIDS epidemic

When the annals of the AIDS epidemic are finalized, the unique role of Dr. Mathilde Krim will be one of the earliest and most influential stories. Dr. Krim died at the age of 91 on January 15th, 2018, having lived a remarkable life. Dr. Krim was born in Switzerland and completed her studies there to become a bench research scientist. She was appalled by the rise of Nazism, including the inhumanity and indifference that she saw taking place in many sophisticated European societies. After the war, she moved to Israel, and became a research scientist at the Weizmann Institute. She was introduced to a visiting delegation of American dignitaries, including Arthur Krim, a prominent New York attorney, who subsequently became the founder of Orion Pictures. In a made‐for‐TV life, Dr. Krim married Arthur, moved to the Upper East Side of Manhattan, and began a laboratory career at the Rockefeller Institute and Memorial Sloan Kettering Cancer Center. In the 1970s, she became interested in understanding the virologic aetiology of some malignancies, and she developed an increasing repertoire of immunologic assays. She was uniquely positioned at the beginning of the AIDS epidemic, since she had the intellect and academic preparation to think about the basic scientific questions posed by the emergence of acquired immunodeficiency, but also understood something about the lives of gay men and the other vulnerable populations who were immediately affected by the epidemic.

Dr. Krim quickly recognized all too familiar patterns of indifference and victimization that were beginning to take shape in response to the growing pandemic, as key populations were identified and stigmatized. She was appalled by the indifference of the Reagan Administration, as the death toll started to mount. Dr. Krim also recognized the importance of educating the public to be scientifically literate about the problems posed by the AIDS epidemic, in order to engage their better instincts, to create a supportive response to address the epidemic. With the investment of substantial personal resources, she created the AIDS Medical Foundation in the early 1980s, which merged with the foundation established by Elizabeth Taylor on the West Coast to become amfAR in 1983. Over more than three subsequent decades, amfAR has been instrumental in providing starter grants to new investigators to investigate key aspects of the epidemic, often initiating discoveries that were subsequently further refined by governmental grants and the pharmaceutical industry, resulting in therapeutic approaches that have saved millions of lives.

From its very outset, amfAR has had a broad vision that included supporting research, people infected and affected by HIV, and reducing stigma and misinformation. Dr. Krim's broad training, compassion, and familiarity with the media universe undergirded amfAR's pillars of community engagement and education, while asking cutting edge questions, using the newest technologies as they emerged. Dr. Krim's charm and panache enabled her to effectively lobby presidents and other world leaders, and to enhance the sense of self‐worth of people living with HIV. Under her leadership, amfAR has been able to cultivate a wide array of committed AIDS researchers, community‐based investigators and activists. Dr. Krim recognized the need for a multi‐faceted approach to address the AIDS epidemic, and behind the scenes helped to support a variety of responses by civil society, ranging from ACT UP to the Treatment Action Group. She remained passionately committed to addressing the epidemic in all of its many facets, with all available tools, in the lab, in the media, in the halls of power.

It is common to ascribe specific changes in history to unique individuals, because it makes for a better narrative, though often there are multiple players and factors that create the sea changes, that in retrospect seem intuitively obvious. Its quite possible that the NIH would have developed a community‐based research programme and a cure initiative without the prodding from Dr. Krim and amfAR, but many of these initiatives always seemed to be “in the making,” not materializing until amfAR took the first step. There are many other examples where it was amfAR's initiative that led to the subsequent investment of substantial resources. In this way Dr. Krim and amfAR have played a catalytic role throughout the epidemic in pushing the field forward. Dr. Krim leaves a dynamic and vibrant legacy, and she will be deeply missed by her family, myriad friends, and those whose lives she touched from around the world.

## Competing interests

None.

## Authors' contributions

KHM conceptualized and wrote the editorial.

